# “Overburdened…Can't Meet Demand”—Experiences of Oral Health Professionals Providing Dental Care to Children With Special Healthcare Needs in South Africa

**DOI:** 10.1111/scd.70227

**Published:** 2026-09-02

**Authors:** Nancy Njoroge, Ansuyah Magan, Mpho M. Molete, Phumzile Hlongwa

**Affiliations:** ^1^ Faculty of Health Sciences Department of Orthodontics School of Oral Health Sciences University of the Witwatersrand Johannesburg South Africa; ^2^ SAMRC/Wits Developmental Pathways for Health Research Unit Faculty of Health Sciences Department of Paediatrics and Child Health School of Clinical Medicine University of the Witwatersrand Johannesburg South Africa; ^3^ Faculty of Health Sciences Department of Oral Biological Sciences School of Oral Health Sciences University of the Witwatersrand Johannesburg South Africa; ^4^ Faculty of Health Sciences Department of Orthodontics School of Dentistry University of Pretoria Pretoria South Africa

## 1 Introduction

Access to routine dental care at primary healthcare (PHC) facilities for children with special healthcare needs (CSHCN) is limited, and referral to a hospital is often necessary [1]. These children have diverse health conditions which require frequent medical and dental care utilizing various health professionals. Specialized dental care services may be required for some CSHCN. Sedation or dental treatment under general anesthesia (GA) is often used to provide dental care to CSHCN. The services include, inter alia, tooth extractions, restorations, endodontics, stainless steel crowns, management of cleft lip and palate, orthodontic treatment, and maxillofacial surgery. These services are provided by oral health professionals (OHPs) working at academic dental hospitals (ADH) or in the private sector.

Globally, the World Health Organization estimates that 1.3 billion people (1 in 6 people) experience profound disability, about 93 million of whom are children below the age of 15 years [2]. In South Africa (SA), the 2011 census reported that about 609 671 (7.5%) of approximately 8.1 million children between 5 and 14 years of age had a disability [3]. However, the report is not only outdated but did not include all CSHCN but rather only those with impairments as described by the WHO classification, which focuses on activity limitations and participation restrictions [4]. Therefore, the lack of information regarding the CSHCN in SA makes it difficult to plan adequate dental care services for these children.

Over the last two decades (2000–2023), epidemiological data on the status of oral health among CSHCN in SA have been limited [5]. The few available studies, mainly conducted in urban centers reported that the prevalence of caries ranged from 22.5% to over 80% in this population [6, 7, 8, 9, 10]. In addition, the majority of dental caries (68%–100%) among the CSHCN was untreated [5], due to barriers in accessing dental care at PHC, and the prolonged waiting time for dental treatment under GA at tertiary hospitals [11]. These factors consequently result in the treatment of the children being largely reduced to the provision of dental extractions at SA's constrained public health facilities [11, 12, 13].

A few other barriers affecting the provision of adequate dental care have been reported in the literature. In Kerala, India, over 80% of the clinics sampled had limited specialized facilities and equipment, while 71% of them lacked physical accessibility for patients with wheelchairs [14]. Furthermore, in Australia, OHPs cited insufficient funding, inadequate facilities and equipment within the public dental system [15] and the lack of adequate support from specialists and other medical health professionals [16]. In addition, other factors such as insufficient knowledge and training of dental professionals were found in Australia [15], India [17], Trinidad [18], and Jakarta [19]. Similar findings to these studies were reported among dentists treating Down syndrome patients in Belgium [20], Brazil [21], and the Balearic Islands [22], pointing to the need for upskilling to effectively manage these patients [23].

Despite efforts to provide school oral health preventative dental services for CSHCN in SA [24], poor oral health persists among these children [5] and may point to other access barriers to dental care. Access to health care is influenced by several factors such as patient, provider and organizational factors [25]. A systematic review focusing on children with disabilities and access to oral health care concluded that barriers such as provider unwillingness, the child's fear of dental treatment, cost of treatment and inadequate dental facilities hindered access to and provision of services [26]. There are limited studies on barriers faced by OHPs while providing services for CSHCN at public health facilities in SA. Understanding how provider and organizational factors impact the provision of dental care to CSHCN could assist in improving access to dental care for these children. Thus, this study aimed to explore the experiences and factors influencing delivery of oral health care for CSHCN in SA from the perspective of OHPs.

## 2 Methods

### 2.1 Study Design

This was a mixed‐methods study. A sequential explanatory design was chosen in which the quantitative data were collected first, followed by qualitative data [27]. The initial quantitative arm consisted of a survey to examine the experiences faced by OHPs in providing dental care to CSHCN at ADH. This was followed by the qualitative arm to explore the factors influencing the delivery of dental care to CSHCN at these hospitals.

### 2.2 Study Setting

All five academic dental hospitals in SA were approached for inclusion in the study. Three dental hospitals are in Gauteng Province, one in the Western Cape and one in KwaZulu‐Natal Province. The hospitals are part of the tertiary health facilities where CSHCN requiring comprehensive treatment are referred from PHC for basic and specialized dental treatment.

### 2.3 Study Participants

The quantitative component of the study included clinical OHPs (general dentists, postgraduate student dentists, dental specialists, dental therapists, and oral hygienists) at participating ADH. The qualitative component of the study consisted of the clinical heads of pediatric dentistry departments at ADH and respondents from the survey who were directly involved in CSHCN management.

### 2.4 Recruitment of Study Participants

All five ADH in SA were invited to participate. For the quantitative phase, participants were recruited by approving ADH via email to the respective administrator. For the qualitative phase, purposive selection of focus group discussion (FGD) participants was obtained from survey respondents. Additionally, heads of pediatric dentistry from respondent ADH were invited by email after institutional consent.

### 2.5 Sampling Strategy

A convenience sample was used for the quantitative phase, targeting approximately 30 OHPs per ADH. This estimate was based on a South African dental school study reporting about 30 full‐time general dentists at one hospital [28]. Thus, the total sample across five ADH was estimated at 150 OHPs.

Purposive sampling was used for the qualitative phase. Survey respondents were invited to the FGD, targeting 8–10 OHPs. Additionally, one head of pediatric dentistry per ADH (*n* = 5) was invited for in‐depth interviews. Sessions were iterative and terminated by the principal investigator (PI) upon data saturation [18].

#### 2.5.1 Inclusion and Exclusion Criteria

Inclusion criteria: The study participants included OHPs working full‐time at participating ADH and clinical heads of pediatric dentistry departments at ADH.

Oral health professionals not working full‐time at the ADH and other clinical heads of department at ADH, visiting lecturers, honorary appointments and part‐time and sessional workers were excluded from the study.

### 2.6 Data Collection

The data comprised of a survey for OHPs at ADH, followed by the qualitative component consisting of focus groups and interviews. The data for the quantitative phase of the study were collected using a structured self‐administered online questionnaire. The questionnaire, which was modified to include perceptions and challenges faced by OHPs at ADH, was based on a previous study that assessed the knowledge and experiences of care for CSHCN among childcare workers in Gauteng, SA [29]. The link to the questionnaire was emailed to OHPs through an administrator at each ADH. Consenting OHPs followed the link provided, completed the questionnaire and submitted it online. A reminder email was sent after 2 weeks to the contact administrator. A final email was sent after 1 month.

For the qualitative arm, the process of data collection comprised two separate components. First, a focus group discussion (FGD) was conducted among OHPs selected from those who had responded to the survey. Five OHPs consented to participate in the FGD. An interview guide was used to allow participants to share their own perspectives and experiences while providing treatment for CSHCN. The FGD took approximately 45 min, after which the participants had no further insights to discuss implying data saturation was obtained. The data from the discussion were transcribed and checked for accuracy by the PI before analysis.

The second component of the qualitative phase involved in‐depth interviews conducted among the clinical heads of pediatric dentistry at ADH. Three of the five clinical heads consented to participate in the study. The interviews were conducted online using a semi‐structured interview guide. Information sought from individual participants included: the average number of CSHCN they see per week, types of services provided, human and infrastructural resources available, collaborations with other dental and medical colleagues in the hospitals, any challenges faced and ways of mitigating those challenges, including future proposals. The interviews were conducted in English for one hour and were recorded and stored in a password‐protected computer. The researcher took additional notes during the interview process.

### 2.7 Data Analysis

The survey questionnaire data were entered into an Excel spreadsheet and imported into STATA Version14 (Stata Corporation Inc., College Station, TX, USA) for analysis. Descriptive analyses were computed using frequencies and percentages to summarize participant demographic data, dental care services provided to CSHCN, types of disabilities among CSHCN receiving treatment, whether participants had received training in special care dentistry, adequacy of training, participants’ perceptions of support from other healthcare professionals, perception of inter‐professionals’ collaboration and perceived barriers faced in the dental care of CSHCN.

For the qualitative data (FGD and interviews), a thematic content analysis was utilized [30]. In addition, the Levesque framework [25] was used to define access to dental care and guide the analyses.

The strategies used to ensure trustworthiness were credibility, dependability, conformability, and transferability [31]. Credibility was ensured by describing the research process. Dependability was assured as OHPs worked at the ADH, where they constantly treated patients, including CSHCN. Counter‐checking with participants during the interviews was done for clarity to enhance dependability. To improve transferability, this study was conducted at a tertiary‐level health setting so that the methodology can be applied to future mixed‐methods studies. Conformability was enabled by ensuring that the interviews were recorded and later transcribed for accuracy, and that the use of direct quotes from participants were used to highlight the themes.

The analysis was conducted as follows: after the audio recordings were transcribed, the transcripts were read several times, and familiarization of the transcripts was undertaken. With each reading, the principal investigator noted similarities or differences in the texts. The codes from the transcripts were identified. Merging of similar codes and assigning of an appropriate name to the codes. The initial coding was done independently by the principal investigator and one of the co‐authors. The two authors then compared the codes and categorized them into themes. The agreed prominent themes were then linked to the Levesque constructs [25]. All the authors reviewed the established codes and themes to check for potential biases before reporting the findings.

### 2.8 Ethical Considerations

The study adhered to the Helsinki Ethical Principles for Medical Research involving Human subjects [32]. Ethical clearance was obtained from the Human Research Ethics Committee (Medical) of the relevant institution (Clearance number: M220266). Written permissions to conduct the study were obtained from each ADH to access the OHPs. Participants of the online survey consented before they completed and submitted the questionnaire. Consent for participation and audio recording was obtained from the key informants and participants of the FGD prior to each session. Participant anonymity was not possible during the discussion, but participant codes were used to anonymize the transcripts. Confidentiality was maintained throughout the reporting of the study findings. Guidelines for the protection of personal data stipulated by the POPIA were observed [33].

## 3 Results

### 3.1 Findings of the OHPs Survey

Fifty questionnaires were completed by OHPs from four ADH (three in Gauteng and one in the Western Cape Province). The majority of the OHPs were female (*n* = 36; 72%), above 35 years (*n* = 40; 80%). Two‐thirds of the OHPs had more than 10 years of clinical experience (*n* = 33; 66%), while 10 (20%) had 5–10 years and 7 (14%) had less than 5 years of experience. Of the total respondents, 19 (38%) were postgraduate student dentists, 16 (32%) were general dentists and 7 (14%) were specialists (three community dentistry specialists, orthodontist = 1, maxillofacial surgeon = 1, oral medicine = 1, and anesthetist = 1), 5 (10%) were oral hygienists and 3 (6%) were dental therapists. Table 1 shows the number of study participants, which highlights the institutional variation across responding ADH.

**TABLE 1:** Study participants by institution and clinical experience.

Characteristics	Postgraduate student dentists *n* = 19 (38%)	General dentists *n* = 16 (32%)	Dental specialists *n* = 7 (14%)	Dental therapists *n* = 3 (6%)	Oral hygienists *n* = 5 (10%)	Total *n* = 50 (100%)
Study center:						
A	7	6	3	0	3	19 (38)
B	5	7	3	2	2	19 (38)
C	4	3	0	0	0	7 (14)
D	3	0	1	1	0	5 (10)
Clinical experience:						
<5 years	4	3	0	0	0	7 (14)
5–10 years	5	3	1	0	1	10 (20)
>10 years	10	10	6	3	4	33 (66)

#### 3.1.1 Medical Conditions Among CSHCN

**FIGURE 1:**
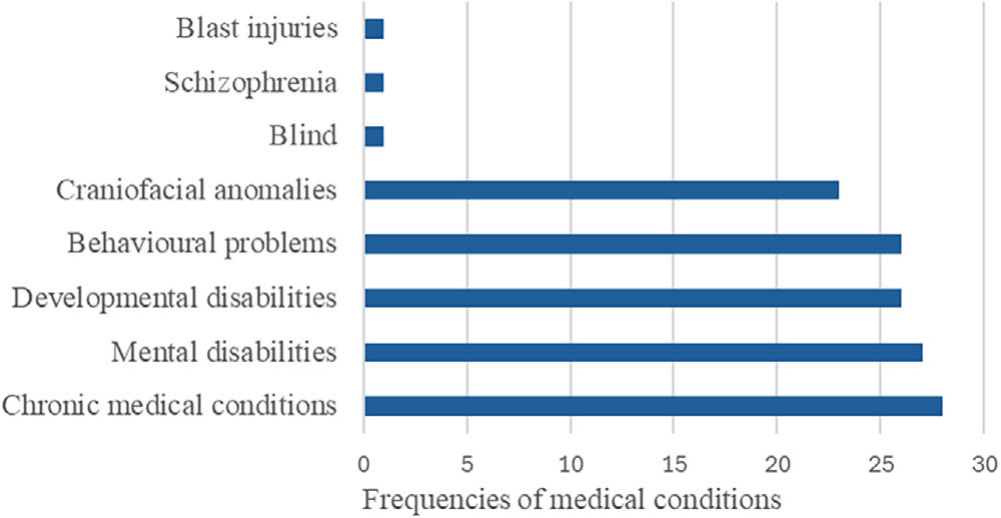
Types of medical conditions among CSHCN who received dental care.

Forty‐three (86%) of the respondents reported that they had treated CSHCN, while 14% referred them to OHPs in pediatric dentistry within the same institution. Figure 1 highlights that the reported medical conditions of the CSHCN treated varied, with the most prevalent being chronic conditions (56%) such as cardiac, immune suppression, and cancer. In addition, over half (54%) comprised CSHCN with developmental disabilities such as autism spectrum disorders, cerebral palsy, and behavioral problems and points to the heterogeneity of CSHCN.

**FIGURE 2:**
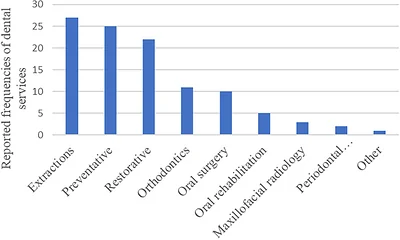
Reported frequencies of types of dental services provided to CSHCN at ADH.

Figure 2 indicates that CSHCN received primarily basic dental care services at ADH, such as extractions (*n* = 27; 54%), preventative (prophylaxis, fissure sealants, fluoride treatment [*n* = 25; 50%], and restorative care [*n* = 22; 44%]). Additionally, there were limited specialty services such as orthodontics and oral surgery provided, while oral medicine/pathology was negligible.

#### 3.1.2 Perceived Challenges Reported by OHPs

**FIGURE 3:**
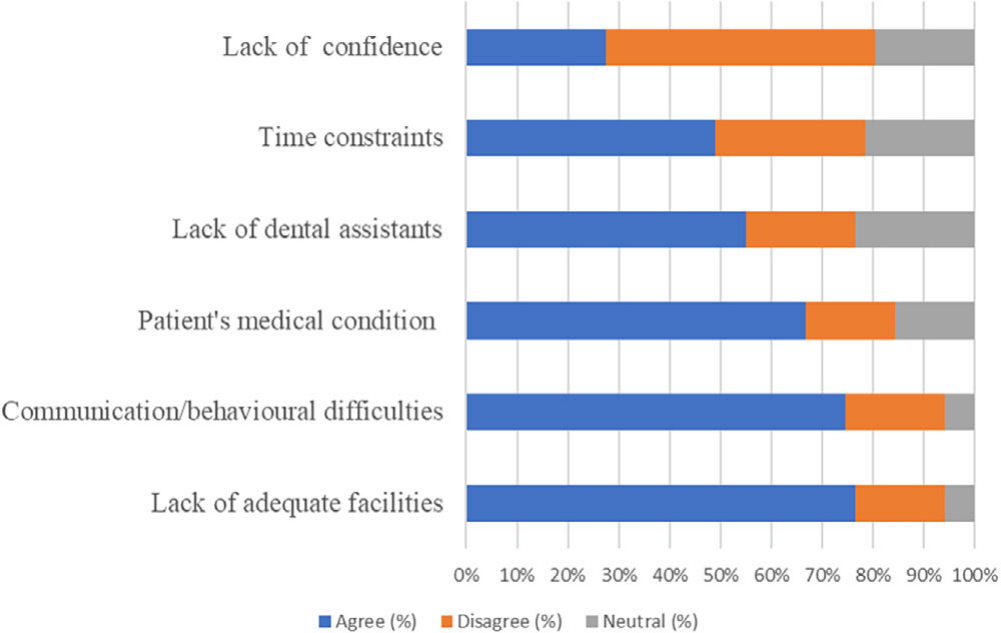
Challenges experienced by OHPs. Agree = OHPs agreed or strongly agreed with statements; Disagree = OHPs disagreed or strongly disagreed with statements; Neutral = OHPs neither agreed nor disagreed with statements.

The survey participants at ADH faced several challenges when providing dental care to CSHCN. Figure 3 highlights that the greatest challenge reported by 76% of OHPs was the lack of adequate facilities especially for theatre. Other concerns, such as the child's behavior and/or communication difficulties, were reported by 74% OHPs while physical/medical condition of CSHCN was mentioned by 66% of the OHPs. Additionally, more than half of the participants (54%) indicated that the lack of dental assistants trained to manage CSHCN was a challenge. Time constraints attributed to the high patient workload and extra time required to manage uncooperative children was cited by 48% as a problem.

**FIGURE 4:**
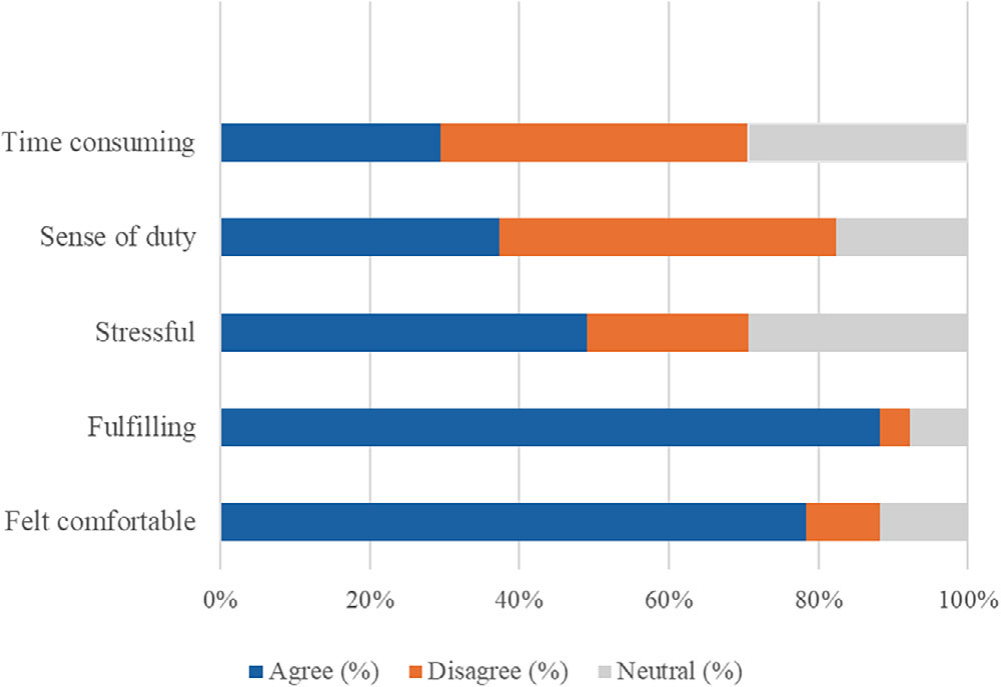
Perceptions of OHPs treating CSHCN.

Overall, despite the challenges, most of the OHPs (78%) felt comfortable and fulfilled (88%) when providing dental care for CSHCN. However, despite the positive sentiments, Figure 4 also highlights that over half of the respondents found it stressful.

Twenty‐six (52%) of the respondents had been trained during their graduate programs to provide dental care for CSHCN. However, of the 50 respondents in the survey, 13 (26%) felt that the training was not adequate, while the same proportion (26%) of OHPs cited it was only satisfactory. Participants mentioned the various ways they improved their knowledge in the dental management of CSHCN, such as learning through experience, self‐initiated online learning programs, continuous education, attending seminars and courses, reading journals, and “learning from each other” or “we learn as we go.”

Twenty‐four (48%) of respondents acknowledged that they received support from other dental specialists such as maxillofacial surgeons and orthodontists while providing treatment for CSHCN. In addition, most of the respondents (98%) felt that inter‐disciplinary collaboration between the medical and dental professionals would improve the quality of dental care services for CSHCN. Respondents suggested that additional support, such as increased collaboration, multi‐disciplinary clinics, a proper patient booking system for GA, increased theatre time allocation, more theatres and sedation facilities would improve dental care for CSHCN.

### 3.2 Findings From the FGD and Interviews

Out of the estimated qualitative (QL) sample of 15, eight (53%) OHPs participated in this section of the study, five for the FGD and three for the interviews. Most of the participants were female (*n* = 7) and dentists (*n* = 6), while two of them were dental specialists (pediatric dentist and prosthodontist).

Four themes emerged from the qualitative analyses. They were service provision, resource constraints, patient affordability, and organizational factors. The themes were linked to the Lévesque framework as depicted in Table 2. There was no link with the Lévesque constructs of acceptability and approachability themes because the study focused on the perspectives of the OHPs and not the patients.

**TABLE 2:** Emerging themes from OHP perceptions on factors influencing the delivery of dental care to CSHCN at ADH.

Themes	Summary findings	Lévesque framework
Service provision	Dental services were largely under GA. There was variation across ADH. Patients travelled long distances to access treatment at ADH. Waiting list challenges were cited. Recommendations included extended clinical partnerships.	Availability: the ability to reach health facilities and professionals timeously.
Resources constraints	Resource constraints such as physical facilities, specialized facilities, finances, human resources, and theatre time affected the availability of services. Lack of pediatric dental specialists. Services were offered by general dentists. Proposed solutions included adequate resources, supplies, and staff training.	Availability: includes the availability of physical facilities and their capacity to generate services.
Patient affordability	Poverty among patients was high. Cost of travel impacted the caregivers’ access to services even though children below the age of 6 years were entitled to free services. Equipping PHC facilities to provide services closer to the patients was proposed to reduce travel costs.	Affordability: includes the economic ability for people to use services and refers to both direct and indirect costs.
Organizational factors	Poor inter‐disciplinary collaborations. Lack of specialty training in pediatric dentistry. Enhanced multi‐disciplinary collaboration was proposed to improve the quality of services.	Appropriateness: relates to the extent to which the services fit the needs of the patient. It includes the type of services provided, quality of services, integration and follow‐up care.

#### 3.2.1 Service Provision

The OHPs highlighted that dental care services for CSHCN were largely conducted under GA. However, the availability of GA services for dental treatment varied across the academic dental hospitals. Participants from three ADH reported that one hospital had three GA morning sessions per week, another had one session while a third did not have any dedicated theatre time for dental treatment. The dental treatment provided also differed across the hospitals and included, inter alia, preventative (prophylaxis, fissure sealants, and fluoride treatment), simple tooth extractions, restorations, and surgical extractions. Specialty services such as orthodontics, maxillofacial, and oral surgery for CSHCN with cleft lip and palate were also rendered from the three ADH. The dental treatments were provided primarily by general dentists working in pediatric dentistry.

“Our staff members should feel comfortable we are general dentistry at the end of the day, and we should be able to manage a paediatric patient adequately.” (KI 1)

Often patients were placed on waiting lists, and pain relief tended to be given priority when patients sought care owing to the high demand.

“We will fast track the patients to do just the extractions so that we can do pain relief.” (KI 2)

“We even get patients from upcountry… so that means that we are overburdened… and we can't meet the demand.” (KI 2)

#### 3.2.2 Resource Constraints

Resource constraints such as inadequate infrastructure, limited finances, and time constraints were reported by the OHPs.

“No, ICU beds for us… There are just no beds for the little ones… and it will also not be good to now mix them together with adult patients that might have probably high infection rates…. No recovery wards.” (KI 1)

“We don't have facilities to perform IV sedation, recovery areas that would be able to help reduce the theatre list and to help with the small emergencies such as extractions… and the clinical ward we don't have enough chairs.” (KI 3)

“We have very limited time in theatre. We only have one morning a week, and which does not allow us to treat a lot of patients because we do full rehabilitation when that child is under general anaesthesia. So, time is an issue.” (KI 3)

Budgetary constraints reported at one hospital resulted in lack of supplies and subsequent cancellation of theatre sessions. This constrained the ability to provide treatment to patients and thus to deliver services.

“We were told that we were at R28 million deficit before we even start. So, we need more than what they gave… So, we already starting on the back foot.” (KI 2)

Time constraints experienced by OHPs affected patient follow‐up care. This was exacerbated by the high number of patients referred for treatment by the surrounding public and private hospitals.

“So, we get a lot more in that we can actually handle… So, the referrals that is quite a problem …” (KI 3)

“Not able to have those opportunities of scheduling appointments, scheduling recalls following a patient on time…” (FGD pp2)

Participants suggested some solutions to mitigate the limited availability of facilities, theatre supplies and resource constraints experienced at the ADH. These included public‐private partnerships, use of non‐profit organizations, and upskilling OHPs in the administration of conscious sedation procedures to minimize their dependency on GA services.

“Umm, we busy now with the talks with uh… private Children's Hospital. That would be a nice private/ public partnership that, you know, we'll have.” (KI 1)

“I've always wanted us to delve into this issue of conscious sedation… sending staff to go do the training on conscious sedation.” (KI 1)

One of the participants suggested that adequate resource allocation from the government was a key solution to financial constraints.

“I think we could do better. Yeah, we could do better. But then government needs to come on board with funding…” (KI 2)

#### 3.2.3 Patient Affordability

The OHPs reported that some caregivers and patients travelled long distances to access the ADH. Thus, the cost of travel was a barrier to accessing dental care. Hence, the OHPs sometimes made concessions for these patients by prioritizing their treatment.

“Sometimes, I have had to request the anaesthetist to see a child who comes from far and is in pain as the parent doesn't have money to come another time.” (KI 2)

“Yeah, that was one mother complaining about the distance … And I realise that some of them come from far, very far. They travel 3 h just to come for that clinic.” (FGD 5)

Furthermore, at one of the hospitals, poor families could not come for the prescribed preventative services (oral health education, diet counselling, prophylaxis, fluoride treatment) as part of the treatment protocols for GA and thus, services offered were solely pain relief.

In addressing patient affordability, OHPs suggested that CSHCN without behavioral issues should have access to basic services closer to their homes to reduce travel costs.

“So, if you find that it's special needs patient who can be cooperative and have that basic preventive care such as prophylaxis, fissure sealants, fluoride application at a local clinic, then it saves them travel.” (FGD pp1)

#### 3.2.4 Organizational Factors

The ADH were largely able to provide dental care to the majority of CSHCN; however, other organizational factors affected the adequacy of services and follow‐up care. These included a lack of specialty training in pediatric dentistry and poor interdisciplinary collaboration.

“We have a specific Cleft clinic that is run by the department of maxillofacial oral surgery, So, the issue of multi‐disciplinary and collaborations may not be written down to say these are the structures, but there's a lot that's happening in that space.” (KI 1)

The nature of collaborations between OHPs who mainly consisted of general dentists with other specialists differed. In some ADH the collaborations with other medical and dental specialists in the dental management of CSHCN were lacking.

“We've got an anaesthesiologist, but we still struggle with collaboration, and they are in‐ house. So, you can't just call him for a consultation, you have to write the referral, but we are in the same building on the same floor down the passage…” (KI 2)

“And what I find most challenging is the team, the multidisciplinary team as some of the specialists are frequently missing from the monthly cleft clinic…” (FGD pp 4)

Another issue raised was the lack of specialty training in pediatric dentistry. In addition, OHPs expressed a lack of support for further training to enhance knowledge and skills on the management of CSHCN.

“And as you know, that paeds (paediatric dentistry) not being a speciality in SA. You can only rely on the people who have special interest in paeds….” (KI 1)

“The last training that we've had on special needs and paeds, it was on our undergrad.” (FGD pp2)

Only one participant indicated that she was trained to provide conscious sedation and was able to train other dental colleagues.

“So, I'm trained, and I train others to use nitrous oxide sedation…hence our staff are well trained to provide sedation but not everybody has a heart for paediatric dentistry.” (KI 3)

To enhance appropriateness of care, some of the suggestions to the organizational issues raised by participants included enhancing multi‐disciplinary teams, coordination of care, support for OHPs training, equipping other public health facilities and introducing specialty training in pediatric dentistry.

“To have more multidisciplinary activities and to also be integrated into the health system.” (FGD pp1)

“I would very much like to see paediatric dentistry become a speciality in South Africa and that would give us more scope and would allow us to do more and to reach more children.” (KI 3)

## 4 Discussion

This mixed‐methods study explored the experiences of OHPs and factors affecting the provision of dental care for CSHCN at ADH in SA. The findings from the quantitative (QT) arm revealed that dental services to CSHCN comprised mainly of basic treatment such as extractions, restorations, polishing, fluoride treatment and some specialty treatment including orthodontics, oral surgery for children with cleft lip/palate. The OHPs in the survey reported that they experienced key challenges that included inadequate physical and specialized facilities (theatre and sedation) to provide dental care for these children. The following themes emerged from the qualitative findings: service provision, resource constraints, patient affordability, and organizational factors. However, the OHPs felt positive and found their work fulfilling. They suggested proposals to alleviate the challenges they faced.

In this study, healthcare availability challenges were demonstrated by resource constraints related to physical and specialized facilities (dental chairs, private rooms, children ward, ICU beds, recovery areas, sedation), finances, lack of specialists, and inadequate theatre time and supplies. This was corroborated by most of the survey participants who highlighted the limited specialized facilities for GA and sedation at ADH. As a result of this limited availability, patients were placed on a wait list for GA while OHPs prioritized pain relief as evidenced by the QT results. Furthermore, caregivers travelled from far to access services. According to Lévesque et al. [25], availability issues include not only the physical facilities such as wheel chair ramps, clinic space, and dental units but also the ability of patients to reach professionals timeously. The lack of specialized facilities has also been reported in other developing countries such as Trinidad, Tobago and India [14, 18].

Financial constraints due to inadequate budgetary allocation affected the availability of dental GA services for CSHCN at ADH in SA. The survey participants who were OHPs reported on the challenges of infrastructure, such as the lack of adequate theatre and sedation facilities due to financial constraints. In other studies, authors have reported on under‐resourcing and low priority for oral health services within the public sector [15, 34, 35]. In SA, the lack of a dedicated budget for oral health affected the availability of consumables and money to repair equipment to provide dental services in Limpopo Province [34]. In Australia, oral health professionals reported that under‐resourcing of the public health system and inadequate equipment and facilities affected dental care provision to individuals with special needs [15]. Similarly, in Rwanda, it was noted that the lack of dental equipment and materials was partly due to the prioritization of other health services such as maternal and child health [35]. Thus, there is a great need for governments to provide adequate financial support for oral health services in the public health system to facilitate access to services, especially for vulnerable children.

Oral health professionals in this study stated that some patients had financial difficulties. They noted that some of the caregivers were from low socio‐economic backgrounds, which affected their ability to afford transport costs to access services. This challenge was linked to Lévesque's affordability construct [25] relating to the economic ability for people to access and utilize services. Other studies have reported similar concerns with regard to patient affordability [18, 36, 37, 38]. In Saudi Arabia, caregivers, especially those from low‐income families, experienced accessibility challenges such as distance to the dental clinic, transportation costs and financial costs [36]. Similarly, in Uganda, challenges with transportation and increased transport costs affected caregivers’ ability to access dental services for children with cerebral palsy [38]. Furthermore, in Trinidad, unaffordability due to low income was cited by caregivers of persons with special needs [18]. This emphasizes the need to provide services closer to the families of CSHCN.

In the study findings, organizational factors at ADH influenced the provision of dental services for CSHCN. These included the lack of specialty training in pediatric dentistry, lack of inter‐disciplinary collaborations, and inadequate OHPs training which impacted the quality of services rendered. In Trinidad, OHPs suggested that inter‐disciplinary collaboration was required to provide holistic care and enhanced quality of life for individuals with special needs [18]. Studies in India, Australia, and Malaysia have cited inadequate training of dentists as a barrier to dental care for CSHCN [14, 15, 17, 39]. Supporting training of both dentists and specialists is key as part of increasing capacity and subsequently addressing improved access to dental care for CSHCN.

Our study settings comprised ADH in SA, and the sample size was based on the estimated number of OHPs providing dental services at the four Dental Schools. Fifty OHPs responded to the survey, the majority working in three out of the four ADHs. Notably, the three ADHs are situated in one of the seven provinces in SA, with implications for representativeness. In addition, the three ADH may have had differences in relation to the availability of GA, human resources, and referral mechanisms. Whereas these organizational differences were present, the findings are likely to reflect the state of the SA public health system in general, including the provinces that were underrepresented. The historical inequalities continue to persist in post‐independent SA, pointing to deeper systemic issues that affect the delivery of health services [40].

The OHPs in this study proposed solutions to the challenges encountered in the dental management of CSHCN. Their suggestions included advanced training of OHPs, private and non‐profit sector engagement, enhanced public‐private partnerships, and increased budgetary allocation and the development of pediatric dentistry as a specialty in SA. Equipping of PHC facilities to provide basic dental services closer to caregivers of CSHCN was also proposed. The success of these proposals to address the challenges experienced by OHPs requires intentional organizational efforts and government involvement. In a US study, partnerships among community organizations and the use of government oral health programs facilitated the provision of oral health services for young CSHCN [41]. The importance of an appropriate policy and government engagement to address broader access issues encountered in dental care for individuals with special needs was highlighted by OHPs in Trinidad and emphasized the essential role of the government in providing financial support [18]. Other factors, such as community dental outreach programs [37], enhanced collaborations of dental and medical health providers, could additionally improve access to dental care for CSHCN. Patients accessing care in multi‐disciplinary settings are also likely to receive regular dental care [42]. Therefore, improved communication and collaborations between different stakeholders such as parents, dental and medical providers, and the establishment of clear referral systems are necessary for timely integrated and holistic care [43].

A key highlight of the study relates to the inadequacy of the undergraduate training in special care dentistry. Additionally, this was contextualized by the qualitative interviews as few opportunities exist for postgraduate or in‐service training in pediatric dentistry. Thus, some of the practical recommendations to address these gaps include curriculum reforms in pediatric dentistry for undergraduate dental students. Structured courses in special care dentistry and practical training in conscious sedation could be used to enhance professional development for OHPs in the public health facilities.

### 4.1 Strengths and Limitations

The use of a sequential mixed‐methods design, combining first the quantitative followed by qualitative arms, provided the breadth and the depth for the study findings. The QT survey enabled us to obtain the broad experiences of OHPs regarding the provision of dental services for CSHCN, while the QL findings from the in‐depth interviews revealed deeper insights into factors linked to the challenges. Triangulation of both QT and QL findings improved the validity of the research. Furthermore, the use of the framework analysis for the QL data helped to summarize the findings and derive more meaningful insights [30]. In addition, the Levesque framework of healthcare access strengthened this study as it enabled us to further report the QL findings in line with some of the access constructs.

Despite the methodological strengths of the study, one of the main limitations was the low number of respondents from the OHPs survey. Additionally, the OHPs were primarily from three of the four ADHs in SA. While the study findings cannot be generalized, they captured the experiences of OHPs in the three Dental Schools which are situated in one province. Notwithstanding the limitations, the QL findings provided deeper and richer information to augment the QT survey. The perspectives and experiences of OHPs rendering services at ADH may have been subjected to some recall bias; however, the multiple data collection approaches utilized, including the focus group and in‐depth interviews, mitigated the impact of the bias.

## 5 Conclusions

The OHPs at ADH experienced challenges related to service provision, resource constraints, low patient affordability and organizational factors that in turn affected the availability of quality dental care for CSHCN.

The study findings have both policy and health systems strengthening relevance for SA. Strengthening PHC level facilities and improving the referral mechanisms could address some of the systemic challenges mentioned earlier in the short‐term. In addition, long‐term strategies include enhanced budgetary allocation to support delivery of oral health services in the public health system. Recognition of Pediatric Dentistry as a specialty in SA form an important part of the broader term recommendations. These measures will collectively enhance access to pediatric oral health services, reduce the referral burden of CSHCN to ADH and promote health equity in line with the government's broader agenda of universal health coverage.

## Author Contributions

N.N. designed the study supervised by P.H. and A.M. N.N. collected and analyzed the data, led the writing as part of her PhD. M.M. assisted with the qualitative data analyses. A.M, P.H., and M.M. edited, revised, and critically reviewed the work. All authors approved the final manuscript.

## Conflicts of Interest

The authors declare no conflicts of interest.
